# Long-Term Oncological Implications of Hormone Replacement Therapy in Women With Hypogonadotropic Hypogonadism: A Propensity-Matched Study

**DOI:** 10.7759/cureus.111721

**Published:** 2026-06-29

**Authors:** Sarah Eidbo, Mitan Nana, Sam King, Maxim Barnett, Ryan Mayo

**Affiliations:** 1 Internal Medicine, Jefferson Einstein Medical Center, Philadelphia, USA; 2 Hematology and Oncology, Jefferson Einstein Medical Center, Philadelphia, USA

**Keywords:** female, hormone replacement therapy, hypogonadotropic hypogonadism, outcomes, trinetx

## Abstract

Introduction

Hypogonadotropic hypogonadism (HH), or secondary gonadal dysfunction, is a result of gonadal failure due to deficiencies in gonadotropin secretion, caused by hypothalamic or anterior pituitary dysfunction. There are multiple forms of HH, with the syndrome divided into congenital, acquired organic, and functional forms. The relationship between HH and oncological processes is complex and multidirectional, and little is known about the relationship between HH and cancer in female patients. Hormone replacement therapy (HRT) can be important for symptom control in many women with HH. However, exogenous estrogen use is additionally associated with a higher risk of both arterial and venous thromboses. There is little information available regarding the impact of HRT on rates of cancers or thromboses in women with HH.

Methods

TriNetX’s US Collaborative Network was utilized to study rates of development of estrogen-associated cancers in female patients at or above 50 years old diagnosed with HH using ICD-10 codes. Adult female patients diagnosed with HH were identified and stratified according to HRT use and ICD-10 codes. HH patients prescribed HRT were matched with a control group of HH patients without HRT based on age, ethnicity, race, and comorbidities. Patients were followed to assess rates of estrogen-associated cancer development at any time after the index event, defined as HH development with or without HRT prescription, or menopause development. Cancers studied included breast, cervical, uterine, ovarian, endometrial, gastric, and colorectal cancer. Development of venous thromboses, including deep venous thromboses (DVTs) and pulmonary emboli (PEs), was also studied. Patients with outcomes prior to the indexing date were excluded from the study.

Results

Following propensity-score matching, we identified 8,293 female patients at or above 50 years old in each cohort, with diagnosed HH, but with and without prescribed HRT. There were no significant differences in rates of breast, cervical, uterine, ovarian, endometrial, or colorectal cancers between HH cohorts. There were no significant differences in rates of DVTs or PEs between HH cohorts.

A cohort of female patients at or above 50 years old with HH and no HRT prescription was propensity-score matched with a cohort of post-menopausal women at or above 50 years old without HH or HRT prescription. There were no significant differences in rates of breast, colorectal, cervical, or ovarian cancers. The postmenopausal cohort demonstrated a significantly increased risk of endometrial cancer (HR 0.246, 95% CI 0.220 - 0.275, p<0.001). The HH without HRT cohort demonstrated a significantly increased risk of gastric cancer (HR 3.150, 95% CI 2.549 - 3.892, p=0.001), acute DVT (HR 1.709, 95% CI 1.595 - 1.831, p<0.001), and acute PE (HR 1.612, 95% CI 1.494 - 1.740, p<0.001).

Conclusions

Our findings indicate that female patients at or above 50 years old with HH may have an elevated risk of venous thromboses and gastric cancer, but reduced risk of endometrial cancer when compared to a cohort of post-menopausal female patients above 50 years old without HRT prescription. No significant differences existed between cohorts with HH with and without HRT prescription. Further study into this population is warranted.

## Introduction

Hypogonadotropic hypogonadism, or HH, is a result of gonadal failure due to deficiencies in gonadotropin secretion, caused by hypothalamic or anterior pituitary dysfunction [1]. It is also known as secondary gonadal failure, indicated by a disruption in the hypothalamic-pituitary axis originating in the hypothalamus or pituitary, rather than testes or ovaries [1]. Low sex steroid levels are seen with inappropriately low or normal gonadotropin levels [1]. There are multiple forms of HH, with the syndrome divided into congenital, acquired organic, and functional forms [1,2]. Congenital forms include syndromes such as Kallmann syndrome, characterized by HH with anosmia or hyposmia [2]. Pituitary macroadenomas, craniopharyngiomas, and infiltrative diseases are included among acquired organic causes of HH [1]. Functional HH, an increasingly common form, is often associated with obesity, type 2 diabetes mellitus, glucocorticoid or opioid use, and anabolic steroid withdrawal [1]. 

The relationship between HH and oncological processes is complex and multidirectional. HH can develop because of malignancy or its treatment but can also serve as a marker of cancer-related physiologic stress or endocrine disruption [3,4]. Further complicating the picture, the low levels of gonadotropin and testosterone often reflect multifactorial processes, including systemic inflammation, poor nutritional status, and direct suppression of the hypothalamic-pituitary-gonadal axis [5]. 

The type of malignancy involved also plays a large role in the relationship between HH and malignancy. Emerging population-based data suggest that in prostate cancer, untreated hypogonadism may be associated with a lower incidence of localized and metastatic disease, though this may reflect confounding effects rather than a causal relationship [6]. Conversely, prostate cancer itself can suppress the gonadal axis, and the androgen deprivation therapy utilized in its treatment can create or worsen preexisting hypogonadal symptoms [5]. Additionally, a recent study noted that lower luteinizing hormone concentrations in men were correlated with elevated risk of testicular germ cell tumor development, though again a causal relationship cannot be established. However, available research primarily centers around malignancies affecting exclusively male patients; little is known about the relationship between HH and malignancy in female patients [7]. 

For female patients whose HH is caused by cancer treatment or occurs during cancer survivorship, hormone replacement therapy (HRT) can be important for symptom control and for long-term health. Published reviews and survivor studies describe HH as a late effect of some malignancies and gonadotoxic therapy, with HRT playing a role in the mitigation of effects such as fatigue, sexual dysfunction, osteoporosis, and delayed development [8]. Therapy is individualized, as multiple factors can influence the decision of whether to prescribe HRT or not. 

In female patients with an intact uterus, HRT can consist of estrogen and progesterone, with many formulations available. Exogenous estrogen can be used for contraceptive measures, as well as postmenopausal or hypogonadotropic symptom management [9,10]. Regardless of its indication, exogenous estrogen has been associated with a higher risk of both arterial and venous thromboses [9,10]. Though the exact mechanism is unclear, estrogen has been demonstrated in previous studies to affect hemostatic pathways [9,10]. This most commonly manifests as deep venous thromboses (DVTs) of the lower extremities [9,10]. There is little data regarding the risk of DVTs or pulmonary emboli (PEs) in women with HH, who could be at a different level of risk due to their differing hormonal profiles and HRT use. 

This study aimed to determine if HRT use among adult women at or above 50 years of age with HH impacted rates of hormonally associated cancers, including breast, ovarian, uterine, cervical, endometrial, gastric, and colorectal cancer compared to adult women at or above 50 years old with HH not taking HRT. It also aimed to determine if HRT use among adult women with HH impacted rates of DVTs. Furthermore, it compared cancer rates and rates of DVTs or PEs among women with HH not on HRT to post-menopausal women without HH or HRT, to determine if HH diagnosis itself carries a different oncologic or thromboembolic risk profile. 

## Materials and methods

Eligibility and cohort definitions 

TrinetX, a database of deidentified health data across multiple large healthcare organizations (HCOs) throughout the country, was enlisted to find patients as per their International Classification of Diseases (ICD)-10 codes. ICD-10 codes corresponding to HH, HRT, and menopause were used to divide adult female patients at or above 50 years old into cohorts, with ICD-10 codes corresponding to outcomes including development of breast, cervical, uterine, endometrial, gastric, and colorectal cancers as well as DVTs and PEs used to compare cohorts. Conditions and corresponding ICD-10 codes are listed in Table 1. Outcomes and corresponding ICD-10 codes are listed in Table 2. 

**Table 1:** ICD-10 Codes for Hypogonadotropic Hypogonadism, Menopause, and Hormone Replacement Therapy International Classification of Diseases (ICD)-10 codes shown are used to identify cases of hypogonadotropic hypogonadism (HH), menopause, and instances of hormone replacement therapy (HRT) use.

Condition	ICD-10 Name	ICD-10 Code
Hypogonadotropic Hypogonadism (HH)	Hypofunction and other disorders of the pituitary gland	UMLS:ICD10CM:E23
	Hypothalamic dysfunction, not elsewhere classified	UMLS:ICD10CM:E23.3
	Hypopituitarism	UMLS:ICD10CM:E23.0
	Disorders of the pituitary gland and its hypothalamic control	UMLS:ICD9CM:253
	Other disorders of the pituitary gland	UMLS:ICD10CM:E23.6
	Disorder of the pituitary gland, unspecified	UMLS:ICD10CM:E23.7
Hormone Replacement Therapy (HRT)	Hormone replacement therapy	UMLS:SNOMED:266717002
	Hormone replacement therapy	UMLS:ICD10CM:Z79.890
Menopause	Menopausal and female climacteric states	UMLS:ICD10CM:N95.1
	Asymptomatic postmenopausal status (age-related) (natural)	UMLS:ICD9CM:V49.81
	Menopausal and other perimenopausal disorders	UMLS:ICD10CM:N95

**Table 2:** ICD-10 Codes for Outcomes International Classification of Diseases (ICD-10) codes used to identify outcomes are listed here. Outcomes included rates of development of breast cancer, ovarian cancer, cervical cancer, uterine cancer, endometrial cancer, gastric cancer, colorectal cancer, deep venous thrombosis (DVT), and pulmonary embolism (PE).

Outcome	Name	ICD-10 Code
Breast Cancer	Malignant neoplasm of the breast	UMLS:ICD10CM:C50
	Malignant neoplasm of the breast of unspecified site, female	UMLS:ICD10CM:C50.91
	Malignant neoplasm of the unspecified site of the unspecified female breast	UMLS:ICD10CM:C50.919
	Malignant neoplasm of the nipple and areola, unspecified female breast	UMLS:ICD10CM:C50.019
	Malignant neoplasm of unspecified site of the left female breast	UMLS:ICD10CM:C50.912
	Malignant neoplasm of unspecified site of the right female breast	UMLS:ICD10CM:C50.911
	Malignant neoplasm of the upper-outer quadrant of the unspecified female breast	UMLS:ICD10CM:C50.419
	Malignant neoplasm of overlapping sites of unspecified female breast	UMLS:ICD10CM:C50.819
	Malignant neoplasm of the central portion of the unspecified female breast	UMLS:ICD10CM:C50.119
	Malignant neoplasm of the upper-inner quadrant of the unspecified female breast	UMLS:ICD10CM:C50.219
	Malignant neoplasm of the lower-outer quadrant of the unspecified female breast	UMLS:ICD10CM:C50.519
	Malignant neoplasm of the lower-inner quadrant of the unspecified female breast	UMLS:ICD10CM:C50.319
	Malignant neoplasm of the axillary tail of the unspecified female breast	UMLS:ICD10CM:C50.619
	Malignant neoplasm of the axillary tail of the left female breast	UMLS:ICD10CM:C50.612
	Malignant neoplasm of the axillary tail of the right female breast	UMLS:ICD10CM:C50.611
Uterine Cancer	Malignant neoplasm of uterus, part unspecified	UMLS:ICD10CM:C55
	Malignant neoplasm of the isthmus uteri	UMLS:ICD10CM:C54.0
	Malignant neoplasm of the fundus uteri	UMLS:ICD10CM:C54.3
	Malignant neoplasm of overlapping sites of the corpus uteri	UMLS:ICD10CM:C54.8
	Malignant neoplasm of uterine adnexa, unspecified	UMLS:ICD10CM:C57.4
	Carcinoma in situ of the cervix uteri	UMLS:ICD10CM:D06
Ovarian Cancer	Malignant neoplasm of the ovary	UMLS:ICD10CM:C56
	Malignant neoplasm of the right ovary	UMLS:ICD10CM:C56.1
	Malignant neoplasm of the left ovary	UMLS:ICD10CM:C56.2
Cervical Cancer	Malignant neoplasm of the cervix uteri	UMLS:ICD10CM:C53
	Malignant neoplasm of the endocervix	UMLS:ICD10CM:C53.0
	Malignant neoplasm of overlapping sites of the cervix uteri	UMLS:ICD10CM:C53.8
	Malignant neoplasm of the exocervix	UMLS:ICD10CM:C53.1
Endometrial Cancer	Malignant neoplasm of the endometrium	UMLS:ICD10CM:C54.1
Gastric Cancer	Malignant neoplasm of the stomach	UMLS:ICD10CM:C16
	Malignant neoplasm of stomach, unspecified	UMLS:ICD10CM:C16.9
	Stomach, NOS	UMLS:ICDO3:C16.9
	Stomach	UMLS:ICDO3:C16
	Malignant neoplasm of the cardia	UMLS:ICD10CM:C16.0
	Malignant neoplasm of the body of the stomach	UMLS:ICD10CM:C16.2
	Malignant neoplasm of the pyloric antrum	UMLS:ICD10CM:C16.3
	Malignant neoplasm of overlapping sites of the stomach	UMLS:ICD10CM:C16.8
	Malignant neoplasm of the fundus of the stomach	UMLS:ICD10CM:C16.1
	Malignant neoplasm of the pylorus	UMLS:ICD10CM:C16.4
	Malignant neoplasm of the lesser curvature of the stomach, unspecified	UMLS:ICD10CM:C16.5
	Malignant neoplasm of the greater curvature of the stomach, unspecified	UMLS:ICD10CM:C16.6
	Gastric antrum	UMLS:ICDO3:C16.3
	Cardia, NOS	UMLS:ICDO3:C16.0
	Body of stomach	UMLS:ICDO3:C16.2
	Overlapping lesion of the stomach	UMLS:ICDO3:C16.8
	Fundus of the stomach	UMLS:ICDO3:C16.1
	Lesser curvature of stomach, NOS	UMLS:ICDO3:C16.5
	Pylorus	UMLS:ICDO3:C16.4
	Greater curvature of stomach, NOS	UMLS:ICDO3:C16.6
	Personal history of other malignant neoplasm of the stomach	UMLS:ICD10CM:Z85.028
Colorectal Cancer	Malignant neoplasm of the rectosigmoid junction	UMLS:ICD10CM:C19
	Malignant neoplasm of overlapping sites of the colon	UMLS:ICD10CM:C18.8
	Malignant neoplasm of the colon	UMLS:ICD10CM:C18
	Malignant neoplasm of colon, unspecified	UMLS:ICD10CM:C18.9
	Malignant neoplasm of overlapping sites of the colon	UMLS:ICD10CM:C18.8
	Malignant neoplasm of the rectosigmoid junction	UMLS:ICD10CM:C19
	Malignant neoplasm of the sigmoid colon	UMLS:ICD10CM:C18.7
	Malignant neoplasm of the ascending colon	UMLS:ICD10CM:C18.2
	Malignant neoplasm of the transverse colon	UMLS:ICD10CM:C18.4
	Malignant neoplasm of the descending colon	UMLS:ICD10CM:C18.6
	Malignant carcinoid tumor of the ascending colon	UMLS:ICD10CM:C7A.022
	Malignant carcinoid tumor of the descending colon	UMLS:ICD10CM:C7A.024
	Malignant carcinoid tumor of the sigmoid colon	UMLS:ICD10CM:C7A.025
	Malignant carcinoid tumor of the transverse colon	UMLS:ICD10CM:C7A.023
	Malignant carcinoid tumor of the large intestine, unspecified portion	UMLS:ICD10CM:C7A.029
	Malignant neoplasm of the hepatic flexure	UMLS:ICD10CM:C18.3
	Malignant neoplasm of the splenic flexure	UMLS:ICD10CM:C18.5
Acute Venous Thromboembolism (DVT)	Acute embolism and thrombosis of unspecified deep veins of the unspecified lower extremity	UMLS:ICD10CM:I82.409
	Acute embolism and thrombosis of unspecified deep veins of the lower extremity	UMLS:ICD10CM:I82.40
	Acute embolism and thrombosis of unspecified deep veins of the left lower extremity	UMLS:ICD10CM:I82.402
	Acute embolism and thrombosis of unspecified deep veins of the right lower extremity	UMLS:ICD10CM:I82.401
	Acute embolism and thrombosis of unspecified deep veins of the lower extremity, bilateral	UMLS:ICD10CM:I82.403
	Acute embolism and thrombosis of other specified veins	UMLS:ICD10CM:I82.890
	Acute embolism and thrombosis of the deep veins of the unspecified upper extremity	UMLS:ICD10CM:I82.629
	Acute embolism and thrombosis of the unspecified femoral vein	UMLS:ICD10CM:I82.419
	Acute embolism and thrombosis of the unspecified tibial vein	UMLS:ICD10CM:I82.449
	Acute embolism and thrombosis of the unspecified popliteal vein	UMLS:ICD10CM:I82.439
	Acute embolism and thrombosis of the unspecified iliac vein	UMLS:ICD10CM:I82.429
	Acute embolism and thrombosis of the unspecified axillary vein	UMLS:ICD10CM:I82.A19
	Acute embolism and thrombosis of the left femoral vein	UMLS:ICD10CM:I82.412
	Acute embolism and thrombosis of the right femoral vein	UMLS:ICD10CM:I82.411
	Acute embolism and thrombosis of the left popliteal vein	UMLS:ICD10CM:I82.432
	Acute embolism and thrombosis of the right popliteal vein	UMLS:ICD10CM:I82.431
	Acute embolism and thrombosis of the deep veins of the right upper extremity	UMLS:ICD10CM:I82.621
	Acute embolism and thrombosis of the deep veins of the left upper extremity	UMLS:ICD10CM:I82.622
	Acute embolism and thrombosis of the left tibial vein	UMLS:ICD10CM:I82.442
	Acute embolism and thrombosis of the right tibial vein	UMLS:ICD10CM:I82.441
	Acute embolism and thrombosis of the femoral vein, bilateral	UMLS:ICD10CM:I82.413
	Acute embolism and thrombosis of the left iliac vein	UMLS:ICD10CM:I82.422
	Acute embolism and thrombosis of the right iliac vein	UMLS:ICD10CM:I82.421
	Acute embolism and thrombosis of the popliteal vein, bilateral	UMLS:ICD10CM:I82.433
	Acute embolism and thrombosis of the deep veins of the upper extremity, bilateral	UMLS:ICD10CM:I82.623
	Acute embolism and thrombosis of the tibial vein, bilateral	UMLS:ICD10CM:I82.443
	Acute embolism and thrombosis of iliac vein, bilateral	UMLS:ICD10CM:I82.423
Acute Pulmonary Embolism	Pulmonary embolism	UMLS:ICD10CM:I26
	Single subsegmental thrombotic pulmonary embolism without acute cor pulmonale	UMLS:ICD10CM:I26.93
	Pulmonary embolism without acute cor pulmonale	UMLS:ICD10CM:I26.9
	Pulmonary embolism with acute cor pulmonale	UMLS:ICD10CM:I26.0
	Other pulmonary embolism without acute cor pulmonale	UMLS:ICD10CM:I26.99
	Other pulmonary embolism with acute cor pulmonale	UMLS:ICD10CM:I26.09
	Saddle embolus of the pulmonary artery with acute cor pulmonale	UMLS:ICD10CM:I26.02

A cohort of adult female patients with HH taking HRT was compared to a cohort of adult female HH patients not taking HRT. These cohorts were propensity-matched according to age, race, and comorbidities. A cohort of adult female patients with HH not taking HRT was compared to a cohort of adult female patients without HH and in menopause. These cohorts were propensity-score matched according to age, race, and comorbidities. Demographics and diagnoses utilized for propensity-score matching are listed in Table 3 and Table 4, respectively. 

**Table 3:** ICD-10 Codes for Propensity-Score Matching Demographics International Classification of Diseases (ICD-10) codes listed here were utilized to propensity-score match cohorts according to demographics, including age, race, and ethnicity.

Demographic	Name	ICD-10 Code
Age	Age at Index	AI
Race	White	2106-3
	Black/African American	2054-5
	Asian	2028-9
	Other Race	2131-1
	Unknown Race	UNK
Ethnicity	Hispanic/Latino	2135-2
	Not Hispanic/Latino	2186-5
	Unknown Ethnicity	UN

**Table 4:** ICD-10 Codes for Propensity-Score Matching Diagnoses International Classification of Diseases (ICD-10) codes listed were utilized for propensity-score matching cohorts according to preexisting diagnoses.

Diagnosis	Name	ICD-10 Code
Endocrine	Endocrine, nutritional, and metabolic diseases	E00-E89
Psychiatric/Neurodevelopmental	Mental, behavioral, and neurodevelopmental disorders	F01-F99
Neurologic	Diseases of the nervous system	G00-G99
Circulatory	Diseases of the circulatory system	I00-I99
Respiratory	Diseases of the respiratory system	J00-J99
Gastrointestinal	Diseases of the digestive system	K00-K95
Skin and Subcutaneous	Diseases of the skin and subcutaneous tissue	L00-L99
Other Clinical	Symptoms, signs, and abnormal clinical and laboratory findings, not elsewhere classified	R00-R99
Other Factors	Factors influencing health status and contact with health services	Z00-Z99

Statistical analysis 

Index events, or the day a patient first met cohort criteria, and time windows, or the time after an index event that an outcome could occur in, were used to analyze results. This study did not specify a time window cut-off, to allow for capturing of outcomes any time after the index event. Survival analysis utilized Kaplan-Meier analysis, with Log-rank testing, proportionality testing, median survival testing, hazard ratios, and survival probability calculated. Patients were censored from analysis if they exited a cohort before the time window ended. 

## Results

HH and HRT 

Following propensity-score matching, 8,293 female patients aged 50 years or older were identified per cohort, stratified as per the prescription of HRT. The average age at index for both cohorts was 64.6 +/-11.3 years. The non-HRT cohort had an ethnic distribution consisting of predominantly White/Caucasian patients (n=6,337, 76.4%), followed by Black/African American (n=1,086, 13.1%), Hispanic/Latino (n=660, 8.0%), and Asian (n=243, 2.9%). Within the HRT cohort, ethnic distribution was similar: predominantly White/Caucasian (n=6,329, 76.3%), followed by Black/African American (n=1,086, 13.1%), Hispanic/Latino (n=655, 7.9%), and Asian (n=243, 2.9%). 

The cohorts demonstrated no significant differences in rates of breast, colorectal, cervical, uterine, ovarian, endometrial, or gastric cancer. They additionally demonstrated no significant difference in rates of acute DVT or PE. Outcomes are summarized in Table 5.

**Table 5:** Hematologic and Oncologic Outcomes of Female Patients with Hypogonadotropic Hypogonadism Outcomes are shown after propensity-score matching for comparisons between the cohort of patients with hypogonadotropic hypogonadism (HH) without hormone replacement therapy (HRT) and the cohort of patients with HH with HRT. Outcomes are also shown after propensity-score matching for comparisons between the HH without HRT cohort and the cohort of patients in menopause without HH. Statistical significance is defined as a p-value at or less than p = 0.05. Cells with asterisks (*) indicate a significantly different risk of this outcome. Cells with hyphens (-) indicate that cohorts were too small to run calculations. Outcomes included development of breast cancer, uterine cancer, cervical cancer, endometrial cancer, ovarian cancer, gastric cancer, colorectal cancer, deep venous thrombosis (DVT), or pulmonary embolism (PE).

	Group 1	Group 2	Breast Cancer	Uterine Cancer	Ovarian Cancer	Cervical Cancer	Endometrial Cancer	Gastric Cancer	Colorectal Cancer	DVT	PE
p-value	HH without HRT	HH with HRT	0.433	0.812	0.585	-	0.667	-	0.84	0.767	0.135
	HH without HRT	Menopause	0.295	0.0001*	0.504	0.841	0.0001*	0.0008*	1.565	0.0001*	0.0001*
HR	HH without HRT	HH with HRT	1.157	0.682	0.62	-	1.176	-	1.291	0.667	0.672
	HH without HRT	Menopause	0.859	0.479*	0.74	0.923	0.246*	3.15*	0.0884	1.709*	1.612*
95% CIs	HH without HRT	HH with HRT	0.914 - 1.464	0.478 - 0.973	0.349 - 1.101	-	0.698 - 1.979	-	0.821 - 2.030	0.564 - 0.789	0.549 - 0.822
	HH without HRT	Menopause	0.805 - 0.916	0.436 - 0.528*	0.643 - 0.852	0.809 - 1.054	0.22 - 0.275*	2.549 - 3.892*	1.38 - 1.773	1.595 - 1.831*	1.494 - 1.740*

HH and endogenous estrogen 

Propensity-score matching identified 77,303 patients per cohort. One cohort had HH without prescribed HRT. The control cohort had been diagnosed with menopause without HH, also without HRT. The average age at index for the HH cohort was 60.7 +/- 7.6 years, compared to the menopause cohort, with an average age at index of 60.8 +/- 12.5 years. The HH cohort was predominantly White/Caucasian (n=44,258, 57.3%), followed by Black/African American (n=13,866, 17.9%), Hispanic/Latino (n=4,709, 6.1%), and Asian (n=4,694, 6.1%). The menopause cohort was similarly predominantly White/Caucasian (n=44,260, 57.3%), followed by Black/African American (n=13,852, 17.9%), Hispanic/Latino (n=4,706, 6.1%), and Asian (n=4,699, 6.1%). 

There was no difference in rates of cervical, colorectal, ovarian, or breast cancer between cohorts. There was a significantly decreased risk of endometrial cancer (HR 0.246, 95% CI 0.220 - 0.275, p<0.001) and uterine cancer (HR 0.479, 95% CI 0.436 - 0.528, p<0.0001) within the HH cohort. There was a significantly increased risk of gastric cancer within the HH cohort (HR 3.15, 95% CI 2.549 - 3.892, p=0.0008), acute DVT (HR 1.709, 95% CI 1.595 - 1.831, p<0.0001), and acute PE (HR 1.612, 95% CI 1.494 - 1.740, p<0.0001). Outcomes are summarized in Table 5.

## Discussion

Oncological risks

Epidemiologic data consistently demonstrate strong associations between circulating estrogen levels and the risk of breast, endometrial, and ovarian cancers [11]. One such study divided postmenopausal women according to estrogen levels, noting an elevated risk of breast and endometrial cancer among the highest estrogen quartile when compared to the lowest estrogen quartile [11]. These risks were increased by two to three times and two to four times, respectively [11]. 

Paradoxically, states of chronic estrogen deficiency, as seen in HH, appear to be protective in the context of hormonal cancers [11]. Strong epidemiological evidence demonstrates that early natural menopause and bilateral oophorectomy substantially reduce lifetime breast cancer risk; menopause before 35 years of age has been associated with a 60-75% reduction in risk [12]. This is biologically plausible, as both estrogens and progesterone act as breast cell mitogens, and their absence reduces the accumulation of carcinogenic mutations in mammary progenitor cells [12,13]. Nonetheless, the hypoestrogenic state carries significant morbidity, including risk of accelerated bone loss, cardiovascular disease, and sexual dysfunction, with severity correlating with earlier age of onset [14]. Furthermore, unopposed estrogen stimulation can disrupt endometrial homeostasis, as estrogen-only therapy has been shown to dramatically increase endometrial cancer risk [15]. This data underscores that the oncologic consequences of HH are not uniform across cancer types. 

Yet, dedicated oncologic outcome data in women with HH remain sparse. Evidence from primary ovarian insufficiency (POI), a HH sharing the core feature of profound hypoestrogenism, demonstrates significantly lower breast cancer mortality compared with age-matched controls consistent with reduced lifetime hormonal exposure [13]. Additionally, mutations in genes governing DNA damage repair and transcription fidelity (identified in up to 43% of women with POI) may independently predispose to cancer risk beyond what is explained by circulating estrogen levels alone, raising the possibility of shared oncogenic mechanisms in congenital HH conditions [16].

Interestingly, our data note no significant difference among cohorts in rates of breast cancer. Cohorts of women with HH stratified as per HRT prescription did not demonstrate a difference in rates of breast cancer. Similarly, the HH cohort without prescribed HRT demonstrated no difference in breast cancer development when compared to the postmenopausal cohort without HH or HRT. This finding could be interpreted as reassuring, as HRT did not impact rates of breast cancer development among women aged 50 years or older with HH. However, we believe that due to the limitations of this study discussed below, further research should explore this relationship before drawing any clinical conclusion. In particular, it is worth noting that there was no significant difference between postmenopausal female patients without HRT prescription and female patients with HH but no HRT prescription. These cohorts were chosen with the intention of studying the effect of endogenous estrogen exposure on oncological outcomes between these cohorts. Given the previous discussion of estrogen's association with various malignancies, one could extrapolate that these two groups would demonstrate a difference in rates of breast cancer, a well-known estrogen-associated malignancy. This was not demonstrated in our study. Further research here is warranted. 

Alongside breast cancer, no difference was noted in rates of cervical cancer development between the postmenopausal cohort without HRT prescription and the HH cohort with no HRT prescription. A dose-dependent relationship between combined oral contraceptive use and rates of cervical cancer development has been previously established, with one review noting that risk doubled when combined oral contraceptives were used for five years or more [16]. Additional studies demonstrated that there was no increased risk of cervical cancer among those using progestin-only contraceptives, further pointing to an estrogen-driven mechanism [17]. However, this finding is not reproduced in this study. Despite a lifelong hypoestrogenic state, the HH cohort without HRT demonstrated no difference in cervical cancer development compared to the postmenopausal cohort. It is important to note, however, that our study did not propensity-score match according to other confounding variables that could affect rates of cervical cancer. These variables include human papillomavirus (HPV) vaccination and parity [18]. It is possible that our cohorts differed significantly in rates of HPV vaccination and in rates of childbirth, especially given the difficulty that women diagnosed with HH can face when attempting to conceive [19]. Further research should focus on cervical cancer rates between these two cohorts, when stratified according to parity and HPV vaccination rates, to better explore any potential difference that could exist. 

No difference in rates of ovarian cancer development was noted between the postmenopausal cohort without HRT prescription and the HH cohort with no HRT prescription. This could be due to a more complex relationship - the relationship between estrogen and ovarian cancer is more multifaceted than in other malignancies studied [20]. Though prior hypotheses had proposed that elevated gonadotropins would stimulate ovarian epithelium and result in elevated ovarian cancer risk, prospective data from previous studies suggested the opposite: lower FSH levels were associated with an elevated ovarian cancer risk, notably in postmenopausal women [20]. Further investigation suggested a relationship that depended more on cancer subtype, which may be a potential confounding variable here [21]. This study grouped all subtypes of ovarian cancer; further research should explore differences according to ovarian cancer subtypes to elucidate whether differences are present. 

Similar to ovarian cancers, colorectal cancer seems to have a complex and multifaceted relationship with estrogen [22,23]. In our study, there was no significant difference between postmenopausal female patients without HRT prescription and female patients with HH and without HRT prescription, suggesting that endogenous estrogen exposure did not influence colorectal cancer development. There appears to be conflicting evidence regarding the role of endogenous estrogen and colorectal cancer development, with some studies noting a protective effect and others noting an increased risk of cancer development [22,23]. Our study noted no difference. Further exploration to elucidate a clearer relationship is warranted.

Though no difference was noted in rates of breast, cervical, ovarian, or colorectal cancer development between the postmenopausal cohort without HRT prescription and the HH cohort with no HRT prescription, there were significant differences between these cohorts in rates of uterine, endometrial, and gastric cancer development. 

The HH without HRT prescription cohort demonstrated a significantly reduced risk of uterine and endometrial cancer development when compared to the postmenopausal cohort without HRT prescription. This finding is consistent with previous research, which demonstrated that unopposed estrogen exposure was the primary driving factor behind two-thirds of endometrial cancers [24]. Previous research has established that estrogen stimulates endometrial proliferation and is typically counterbalanced by progesterone, inducing endometrial shedding and menstruation [24]. It would follow that a cohort with a lifetime of endogenous estrogen exposure would have a higher risk of endometrial and uterine malignancies than a cohort with a lifetime of hypoestrogenism. However, further research should focus on determining if differences exist between these cohorts when more specific cancer subtypes are explored. Further research should additionally stratify for other sources of endogenous estrogen, such as adipose tissue [24].

Gastric cancer is unique among the malignancies studied here, as estrogen exposure appears to confer a primarily protective benefit based on previous epidemiological research [25,26]. Evidence has consistently demonstrated that longer endogenous estrogen exposure is associated with reduced rates of gastric cancer development, which is further demonstrated in this study [25,26]. The HH without HRT cohort demonstrated an increased risk of gastric cancer development compared to the postmenopausal cohort without HRT, which could be due to differences in lifetime endogenous estrogen exposure. 

This study, as noted above, demonstrated no significant difference in rates of cancer development between cohorts with HH stratified according to HRT prescription. Alongside breast cancer development, there was no difference between cohorts in the development of uterine, ovarian, endometrial, cervical, gastric, or colorectal cancers. This finding is initially reassuring, as exposure to HRT appears to confer no additional risk of developing these malignancies in women with HH. However, our study is not without limitations. No dose-dependent stratification was performed in this study to further evaluate the effect of HRT on cancer development, as it was not available within the database utilized for this study. Though cohort sizes were robust and propensity-score matching was thorough, it is possible that confounding variables were overlooked. HH is a rare condition, though with multiple subtypes as discussed previously. This study opted to include all subtypes of HH. Further studies should investigate whether more specific subtypes of HH demonstrate similar findings or if oncologic outcomes differ based on subtype. 

Thromboembolic risks

In addition to the oncological findings above, our study sought to determine differences in the female HH population regarding the development of venous thromboemboli, including DVTs and PEs. 

Exogenous estrogen has been repeatedly demonstrated to significantly increase the risk of DVT development, with the magnitude of risk depending on multiple factors: dose, route of administration, and progestin co-administration among them [27]. Estrogen is believed to induce a prothrombotic state through multiple mechanisms, including decreasing levels of natural anticoagulants, increasing the production of procoagulant factors, and inducing the development of acquired activated protein C resistance [27].

Prior evidence has shown that patients taking oral estrogen have the highest risk of DVT development, likely due in part to the first-pass hepatic metabolism's effect on coagulation factor synthesis [28]. Transdermal estrogen formulations did not see this development, likely due to their avoidance of first-pass hepatic metabolism [28]. 

Interestingly, our data demonstrate no difference in rates of DVT or PE between cohorts with HH but with and without HRT prescription. Multiple factors could contribute to this finding. HRT currently recommended for women with HH primarily consists of transdermal formulations, as these are demonstrated to have less hypercoagulable effect due to bypassing first-pass hepatic metabolism [29]. Multiple formulations of HRT exist, and among women with HH who may be taking oral HRT, many are likely taking a combination of estrogen and progestin to offset the oncological risks associated with unopposed estrogen in premenopausal women [29]. This relationship should be further explored with research stratifying according to HRT formulation, as our study was unable to do so. 

There was a significantly increased rate of DVT and PE in the HH without HRT cohort when compared to postmenopausal women without HRT, however. This finding is paradoxical on the surface, seeming to suggest that the lifelong hypoestrogenic state of women with HH without HRT could place these patients at a higher risk of DVT or PE. It is possible that many patients with HH were mistakenly coded as not having a prescription for HRT. However, it is also possible that this finding could reflect the many consequences of untreated HH in women. Unlike women without HH, there is no alternative treatment for premenopausal women with HH, as prolonged estrogen deprivation has been demonstrated to have severe health consequences [30]. Though exogenous estrogen is associated with increased hypercoagulability, it is possible that this hypercoagulability is outweighed by the multifactorial consequences of hypoestrogenism over a long period of time, as seen in the cohort of female patients with HH and no HRT prescription. Further research into this finding should explore the risk profile of this cohort in relation to other consequences of hypoestrogenism. 

Limitations 

This study has several limitations that necessitate discussion. One limitation is its reliance on TriNetX data, which further relies on ICD-10 coding. If conditions are coded improperly, this could serve as a potential source of error. If the HCOs that are queried do not all respond, this serves as another potential source of error by possibly not adequately reflecting true condition or outcome rates. Relying on ICD-10 coding data further limits cohort propensity-score matching, as evidenced above in our discussion of HRT. Though patients are coded within the database as having prescriptions for HRT, it was not possible to further stratify according to formulation or dosage, both aspects that contribute to oncological and hematologic risks based on previous studies. 

## Conclusions

These findings demonstrate that women at or above 50 years of age with HH diagnosis may have a different risk for hormonally associated cancers including uterine, endometrial, and gastric cancer compared to postmenopausal women at or above 50 years old without HH diagnosis. There may additionally be a difference in risk of DVT or PE among women with HH diagnosis compared to postmenopausal women without HH. Further research should focus on exploring the relationship between these risks and HRT, with different doses, formulations, and lengths of adherence to these medications serving as initial areas to study. 
